# Total aortic arch replacement under intermittent pressure-augmented retrograde cerebral perfusion

**DOI:** 10.1186/1749-8090-5-97

**Published:** 2010-11-02

**Authors:** Hiroshi Kubota, Kunihiko Tonari, Hidehito Endo, Hiroshi Tsuchiya, Hideaki Yoshino, Kenichi Sudo

**Affiliations:** 1 Department of Cardiovascular Surgery, Kyorin University, Tokyo, Japan; 2 Department of Cardiology, Kyorin University, Tokyo, Japan

## Abstract

Kitahori, Kawata, Takamoto et al. described the effectiveness of a novel protocol for retrograde cerebral perfusion that included intermittent pressure augmentation for brain protection in a canine model. Based on their report, we applied this novel technique clinically. Although the duration of circulatory arrest with retrograde cerebral perfusion was long, the patient recovered consciousness soon after the operation and had no neurological deficit. Near-infrared oximetry showed recovery of intracranial blood oxygen saturation every time the pressure was augmented.

## Background

To prolong the safe limits of conventional retrograde cerebral perfusion (RCP), Kitahori, Kawata, Takamoto et al. assessed a novel protocol, intermittent pressure-augmented retrograde cerebral perfusion (IPA-RCP), in a canine model [1-3]. This new protocol was clinically applied to a 51 year-old-male with a diagnosis of acute aortic dissection. Near infrared oximetry showed recovery of intracranial blood oxygen saturation during the pressure augmentation. Although duration of RCP was long, the patient recovered consciousness 30 min after the operation free of any neurological deficit after total arch replacement.

## Case presentation

On July 24, 2006, a 51 year-old-male with a diagnosis of acute aortic dissection (DeBakey I, Stanford A) was transferred to our hospital from a nearby hospital, and emergency operation was performed the same day. The pericardium was opened through a median sternotomy and a cardiopulmonary bypass was established by cannulations the inferior and superior venae cavae and the right femoral artery. Circulatory arrest with retrograde cerebral perfusion was commenced when the patient's tympanic temperature reached to 18.0°C. A large longitudinal intimal tear was present in the greater curvature of the aortic arch, and it ended just proximal to the left subclavian artery. The aorta was transected between the left common carotid artery and the left subclavian artery. The aorta was reinforced with two Teflon felt strips, and a four-branch 24-mm graft was anastomosed. After anastomosis of the left common carotid artery, the graft was clamped, and antegrade perfusion via a side branch and rewarming were started. The brachiocephalic artery was then anastomosed and perfused. Finally, the proximal anastomosis was performed, and the aortic clamp was released. Weaning from the cardiopulmonary bypass was achieved smoothly.

### Retrograde cerebral perfusion

Conventional retrograde cerebral perfusion (RCP) with 15 mmHg of superior vena cava pressure was performed first, and 30 min later, when the anesthesiologist alert that near-infrared oximetry showed a low value under 50%, we converted to the intermittent pressure augmented retrograde cerebral perfusion (IPA-RCP) method with superior vena cava pressure increased to 45 mmHg. The intervals and durations of the augmentations were irregular, because when the backflow from the cervical branch disturbed the anastomosis, the pressure decreased expediently. The maximum duration of augmentation was limited to 30 sec. The circulatory arrest time, conventional RCP time, IPA-RCP time were 85 min, 30 min, and 55 min, respectively, and a total of 10 augmentations were performed. Intracranial regional oxygen saturation (rSO_2_) was measured with a TOS-96 brain oximeter (TOSTEC Co., Ltd. Tokyo, Japan).

## Results

**Figure 1:**
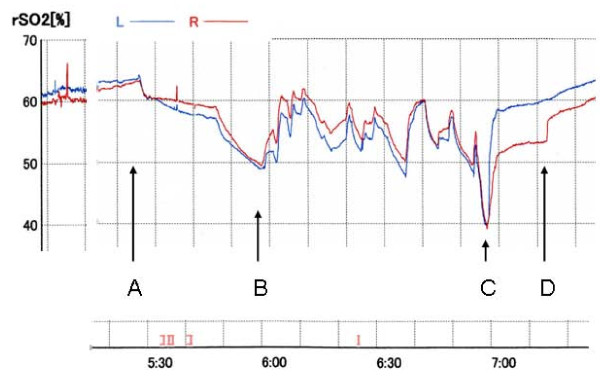
**rSO**_**2 **_**during deep hypothermic circulatory arrest**. L: left rSO_2_, R: right rSO_2_. Initial 30 min of conventional retrograde cerebral perfusion (RCP), rSO_2 _gradually declined. When intermittent-pressure-augmented (45 mmHg) retrograde cerebral perfusion (IPA-RCP) was induced, rSO_2 _rose. The maximum duration of pressure augmentation was limited to 30 sec. A total of 10 augmentations at irregular intervals were tried. A. Start of deep hypothermic circulatory arrest and conventional RCP. B. Start of IPA-RCP. C. Final dip: Start of the antegrade perfusion to the left common carotid artery, and the left subclavian artery via graft branch. D. Start of antegrade perfusion via the brachiocephalic artery.

Prior to the anesthesia, the rSO_2 _was 61% (Left) and 60% (Right). At the beginning of the cardiopulmonary bypass, the rSO_2 _was 55% (Left) and 56% (Right). At profound hypothermia, the rSO_2 _was 64% (Left) and 63% (Right), it gradually decreased to 49% (Left) and 50% (Right). After commencing the IPA-RCP, the rSO2 rose to around 60% at every augmentation, but it decreased when the augmentation ceased. Just after the resuming antegrade perfusion via a side branch of the graft, the rSO_2 _decreased to 40%, then recovered smoothly (Figure 1)_. _The rSO_2 _on the right side recovered in a stepwise manner. The patient recovered consciousness 30 min after the operation free of any neurological deficit and the postoperative course was uneventful.

## Conclusions

RCP by augmentation of CVP to 15 to 20 mmHg is routinely used in our institute for the additional brain protection during deep hypothermic circulatory arrest because much evidence has been accumulated to suggest an increased risk of perfusion-induced brain injury associated with RCP, especially when continuously high RCP pressures are used [4]. However, there is a safety limit of the deep hypothermic circulatory arrest duration because it cannot open all intracranial vessels but partially. To overcome this drawback, Kitahori, Kawata, Takamoto et al. developed a new intermittent pressure augmentation method in which CVP is intermittently increased to 45 mmHg [1-3]. They used a canine model, and showed that the retinal vessels were effectively dilated at an augmented pressure of 45 mm Hg (arteries, 107% + 3% of control veins, 114% + 3% of control), whereas when antegrade selective cerebral perfusion was used, the retinal vessels were smaller than the corresponding preoperative vessels. They concluded that the intermittent pressure augmentation allows an adequate blood supply without injuring the brain and provides adequate neuroprotection equivalent to that provided by antegrade cerebral perfusion. In the canine model, they administered the RCP through the maxillary vein to overcome the drawbacks of jugular vein valves to reach directly the cranial veins. In the majority of humans, as de Brux et al. described, the jugular vein had competent valves and it is hypothesized that the RCP gains the brain through a collateral network of veins (azygos, intercostal, medullary and vertebral veins). The usefulness of higher perfusion pressure could be either to distend the valves or more probably to increase the pressure in the collateral vein network to improve cerebral oxygenation [5]. Thus, the clinical effectiveness of the IPA-RCP through a cannulae inserted to the SVC is unknown field. We examined the effect of the IPA-RCP by measuring rSO_2 _which represents the brain blood perfusion. Although only the anterior part of the brain rSO_2 _is assessed by a TOS-96 brain oximeter, because most attenuation of near-infrared light in human cerebral tissues is due to absorption by deoxyhemoglobin and oxyhemoglobin, brain tissue is suitable for determination of rSO2. Only determination of rSO2 is an easily available method to assess the real-time adequacy of cerebral perfusion during deep hypothermic time-restricted aortic arch surgery [6].

At first, we planned to perform the operation on our patient using conventional RCP. However, because the rSO2 declined to 49%, the duration of circulatory arrest time was expected to exceed 60 min due to the fragile aortic wall to reinforce and deep distal anastomosis, we applied the intermittent pressure augmentation technique for the first time. According to the original report, the central venous pressure was controlled at 15 mm Hg and it was augmented to 45 mm Hg quickly and then decreased again to the baseline level of 15 mmHg as soon as it reached 45 mm Hg every 30 seconds. However, the same protocol is difficult to apply clinically because backflow from the three arch vessels increased and disturbed the anastomosis when CVP was augmented. CVP was decreased to 15 mmHg expediently. Although the optimal duration of pressure augmentation during deep hypothermic circulatory arrest in clinical settings is unknown, to prevent the brain edema, the maximum duration of pressure augmentation that we set was 30 sec.

Along with every pressure augmentation, rSO2 showed immediate recovery up to 60% and it decreased when the augmentation ceased. The essential effect of IPA-RCP may not only be a temporary increase in rSO2 but elevation of the declining curve during RCP. Our preliminary randomized comparative study in clinical aortic arch replacement cases of IPA-RCP (n = 10) and standard RCP (n = 10) showed that the interval from the end of the operation to full awakeness of the IPA-RCP group was 85 ± 64 min. in contrast with 310 ± 282 min. in RCP group (p < 0.05) accompanying with the rSO2 decline ratio 60 min after the initiation of the IPA-RCP group was 13.1 ± 3.7% in contrast with 24.5 ± 13.1% in RCP group (p < 0.05). There was no significant difference of the used amount of the anesthetic agent. It may support the "bottom raising effect" of this new protocol.

Just after the resumption of antegrade perfusion, the rSO_2 _decreased to 40%, but then recovered smoothly. We named this phenomenon the "final dip". When we use RCP, the final dip always appears just after the resumption of antegrade perfusion. This phenomenon may represent wash out of deoxygenated blood that remained and did not circulate in the brain despite the performance of retrograde cerebral perfusion. The stepwise recovery of the rSO_2 _of the right side may mean that the resumption of antegrade perfusion via the left arch branches was insufficient to wash out the remaining blood in our patient. In conclusion, this novel protocol may have some advantages over conventional RCP. Because it is difficult to verify the efficacy of IPA-RCP by quantitative analysis, accumulation and analysis of data e.g. measurement of the concentration of Tau proteins in the CSF, comparison of the pre- and postoperative cognitive function, measurement of the diameters of the retinal vessels during IPA-RCP may demonstrate the advantages of this new method of brain protection [7].

## Competing interests

The authors declare that they have no competing interests.

## Authors' contributions

HK, KT, HE, HT conceived of the study, and participated in its design and coordination. HY and SK participated in the sequence alignment. All authors read and approved the final manuscript.
